# Harmonization of the Practice of Independent Ethics Committees in Italy: *Project E-Submission*

**DOI:** 10.1371/journal.pone.0048906

**Published:** 2012-11-07

**Authors:** Gianfranco De Feo, Giacomo Chiabrando, Nunzia Cannovo, Antonio Galluccio, Carlo Tomino

**Affiliations:** 1 National Cancer Institute, Naples, Italy; 2 S. Orsola University Hospital, Bologna, Italy; 3 Ethics Committee of “Federico II” University, Naples, Italy; 4 Italian Medicines Agency, Rome, Italy; Istituto Superiore di Sanità, Italy

## Abstract

**Aim:**

The high variability of “centre-specific” documentation required by Independent Ethics Committee (IEC) plays a role in the time required for activation of participating centres of multicentre clinical trials. This study (a) provides a picture of the different activities, structural requirements and resources dedicated to the operation of the local IEC in Italy; (b) defines a detailed list of “centre-specific” documents considered as essential by the IEC for issuing its opinion and (c) suggests a “single document” to reduce the variability of the “centre-specific” documents required by the IEC.

**Methodology:**

Two surveys were conducted through the portal of National Monitoring Centre of Clinical Trials (https://oss-sper-clin.agenziafarmaco.it/). The first survey focused on the local IEC resources and on the “centre-specific” documentation that local IEC required from the Sponsor and local Principal Investigator (PI). The second focused on “single document” required in the form of statements from the Sponsor and the PI. Answers were discussed and extended during regular scheduled teleconferences and plenary meeting.

**Principal Findings:**

From 22/07/2009 to 15/12/2009, and from 19/04/2010 to 14/05/2010, 131 and 125 IECs responded to the first and the second surveys, respectively. 67% and 51% of IECs consider the structural requirements and the staff dedicated to the activity of the IECs as sufficient, respectively. Most of the IECs consider the “centre-specific” documentation as necessary for issuing the opinion, and a high percentage of IECs consider the proposed documentation as acceptable in substitution to any other “centre-specific” documentation already in use.

**Conclusions:**

The harmonization of IECs practice in Italy is the first step to facilitate multicentre clinical trials. Similar efforts should be directed to reduce the total number of IECs and to standardize clinical trials approval procedures, focusing on administrative procedures as well.

## Introduction

The European Directive 2001/20/EC legally ensured the implementation of the principles of good clinical practice in clinical trials on medicinal products in Europe [1]. The Directive establishes, in all Member States, specific provisions regarding clinical trials conduction, including multicentre trials on human subject involving medicinal products and harmonizes the practice of Independent Ethics Committees (IEC) and administrative provisions.

In Italy, the European Directive was implemented by the Legislative Decree 211/2003 [2] and regarding the multicentre clinical trials activation procedure, the Legislative Decree 211/2003 considers three subsequent phases: first, the clinical trial application submission to the IEC of both the coordinating and the participating centres as well as the administrative agreement submission to the Competent Authority of participating centres; second, the issuance of the “single” opinion by the IEC of the coordinating centre and, in case of positive opinion, the acceptance or refusal by the IEC of each participating centre; third, in case of acceptance, the trial contracts signature between the coordinating and each participating centre.

A survey of 134 Italian IECs, reporting on a single trial, demonstrated a large variability of the clinical trial application procedures, both for the number and the format of required documents. The number of necessary documents ranged from 6 to 21; 57% of the IECs required at least one personalized document, with a number of hardcopies ranging from 6 to 249. Furthermore, 26.9% of IECs asked for e-mail or CD-ROM submission (number of copies ranging from 1 to 15) in addition to the paper version [3].

The scenario could have changed in Europe thanks to the publication of the detailed guidance about the clinical trial authorisation request to competent authorities [4], on the format of the application and the documentation to be submitted for IEC opinion [5], and on investigational medicinal products and other medicinal products [6]. These guidelines defines all the operative procedures and the required documents for a clinical trial authorisation; it also introduces the CTA form, with the unique aim of identifying the clinical trial, the organisations and the key individuals responsible for the conduction of the trial.

However, a recent report on the Procedure for the Ethical Review of Protocols for Clinical Research Projects in Europe and Beyond [7], reported most European Countries to require specific national CTA forms, and that some IECs to require further documentation than the one requested by the guidelines.

In Italy, these guidelines were implemented in December 2007, with the Ministerial Decree on clinical trial application (MD-CTA) [8].

Regarding the impact of MD-CTA on the time required for obtaining IEC opinion and contract signature, a study showed an encouraging trend towards reduced time spent for activating oncology non-profit studies in Italy. However, the MD-CTA doesn’t seem to impact the time required for administrative agreement signature. Hence, the median time for obtaining IEC opinion has been shorter (median 2.4 *vs* 4.1 months) than before of MD-CTA’s introduction, while the median time to sign the administrative agreements has not changed yet (median 3.6 and 3.8 months, respectively). Time to trial activation is critical for clinical research. Therefore, major efforts should be made to reduce and standardize procedures concerning the approval of clinical trials [9].

The Italian Medicines Agency (AIFA) promoted, since December 2008, a project for electronic submission of all the necessary documentation concerning a clinical trial (*project e-submission*). The project involves IECs, Italian National Health Institute (ISS), profit and non-profit Sponsors and Contract Research Organizations (CRO). One of the objectives of the project was to define a detailed list of “centre-specific” documents required by the IEC in addition to the files defined by the MD-CTA (CTA form and lists documentary Ia and Ib) that the IECs, in accordance with their procedures, consider as essential for the issuance of the opinion.

This study (a) provides a picture of the different activities, structural requirements and resources dedicated to the operation of the local IEC in Italy; (b) defines the detailed list of “centre-specific” documents considered essential by the local IEC for the issuance of the opinion and (c) tries to suggest a “single document” to reduce the variability of the “centre-specific” documents required by IECs.

## Methods

Two surveys were conducted through the portal of National Monitoring Centre of Clinical Trials (OsSC) (https://oss-sper-clin.agenziafarmaco.it/). The OsSC was created by the AIFA in 2000, in order to supervise the clinical trials of investigational medicinal products conducted in Italy. Authorized users (Sponsor, CRO, IECs, ISS and AIFA) can access the OsSC in order to: (a) prepare and submit documentation to obtain the IEC’s opinion and the Competent Authority’s authorization; (b) communicate the IEC’s and the Competent Authority’s decisions; (c) notify the start and completion of clinical trials and the publication of trial results.

The first survey, in the form of questionnaires was focused on three documents:

1. Resources of the local IEC;
2. “Centre-specific” documentation required from the Sponsor or CRO;
3. “Centre-specific” documentation required from the local principal investigator (PI);

All IECs, in Italy, were asked to define the following:

- The activities, structural requirements and staff resources dedicated to the operation of the IECs;
- The “centre-specific” documentation they consider necessary for the issuance of IEC opinion;
- Until all documentation is submitted electronically, the modality they consider most appropriate for sending the “centre-specific” documentation.

Using the results of the first survey, a “single document” was developed to establish, at the national level, the “minimum” elements, in addition to the CTA, required by IECs. A second survey was conducted to obtain feedback on the developed document.

The second survey, in the form of questionnaires, was focused on:

1. A “Single document” required in the form of statements from the PI (Form 1).
2. A “Single document” required in the form of statements from the Sponsor (Form 2).

The IECs, that answered the first survey, were asked to answer the following question:

- Are Form 1 and Form 2 acceptable to the IEC, in place of any other “centre-specific” documentation already in use?

Answers were discussed and extended during regular scheduled teleconferences and plenary meeting.

## Results

From July 22^th^, 2009 to December 15^th^, 2009, out of 268 accredited IECs, 131 (49%) answered the survey. Table 1 shows the geographic distribution of the IECs that answered the survey. The IECs that responded issued 617 of the 851 “single opinions” (72.5%) released in 2008.

**Table 1:** Distribution of the IECs by geographic location.

	Total IECs accredited on 2008		IECs that answered the first survey	
	No.	(%)	No.	(%)
Location				
North	110	(41.1%)	73	(66.3)
Centre	58	(21.6%)	30	(51.7)
South or islands	100	(37.3%)	28	(28.0)
**Total**	**268**	**(100.0%)**	**131**	**(48.9)**

### 1. Resources of the Local IEC

**Figure 1:**
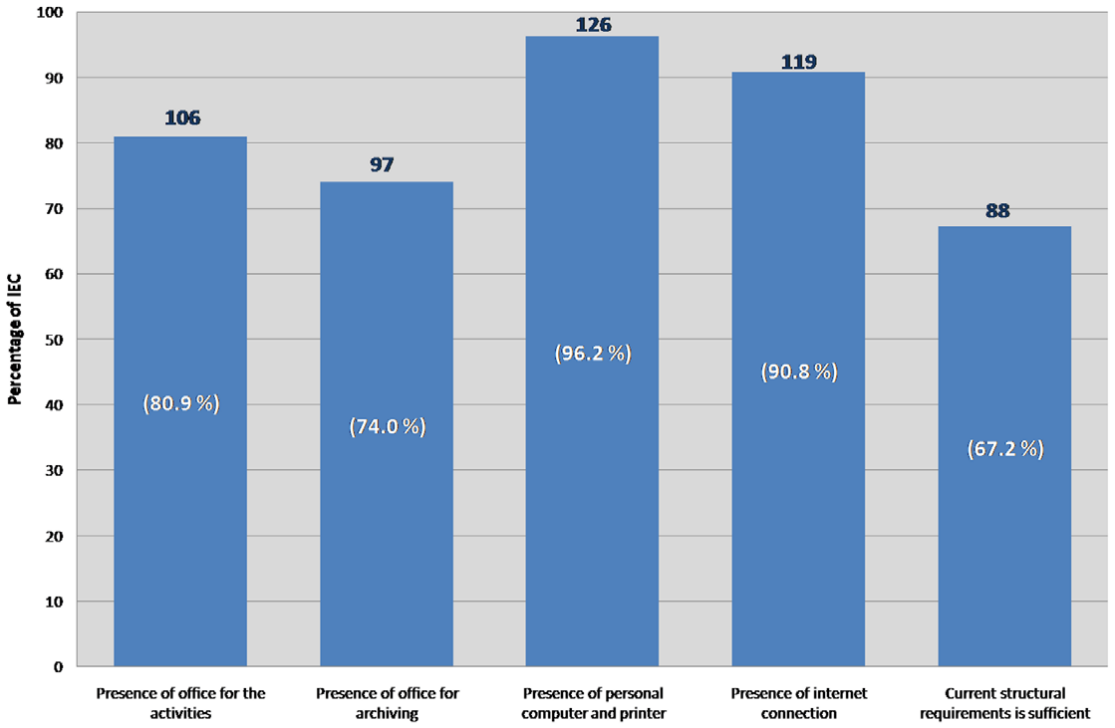
Structural requirements dedicated to the technical-scientific secretariat of IECs. IECs that answered the first survey are 131 out of 268 accredited.

Regarding the structural requirements dedicated to the technical-scientific secretariat of the IECs, 88/131 (67.2%) consider the current structural requirements as sufficient. In particular, 80% have a dedicated office to the activities of the technical-scientific secretariat of the IECs and to archive any study documentation, and most of them are equipped with at least one personal computer and an internet connection (Figure 1).

Regarding the staff resources dedicated to the operation of the technical-scientific secretariat of the IECs, only 66 (51.2%) IECs consider the current resources as sufficient; Table 2 shows that, on average, less than one equivalent unit of scientific staff and only one equivalent unit of administrative staff are dedicated to the activities of the technical-scientific secretariat of the IECs.

**Table 2:** Staff resources dedicated to the technical-scientific secretariat of the local IEC.

Answer (n = 131)	No. (%)
Presence of the OsSC referent for the “study non-profit”	102 (77.9)
Current staff resources, sufficient:	66 (51.2)
Scientific staff - equivalent unit full or part-time	103
Administrative staff - equivalent unit full or part-time	133

### 2. “Centre-specific” Documentation Required of the Sponsor or CRO

Ninety-six (73.3%) IECs consider the “centre-specific” documentation requested of the Sponsor or CRO as necessary. Among these, seventy-one (74%) believe that electronic submission is the most appropriate modality for sending the “centre-specific” documentation.

**Figure 2:**
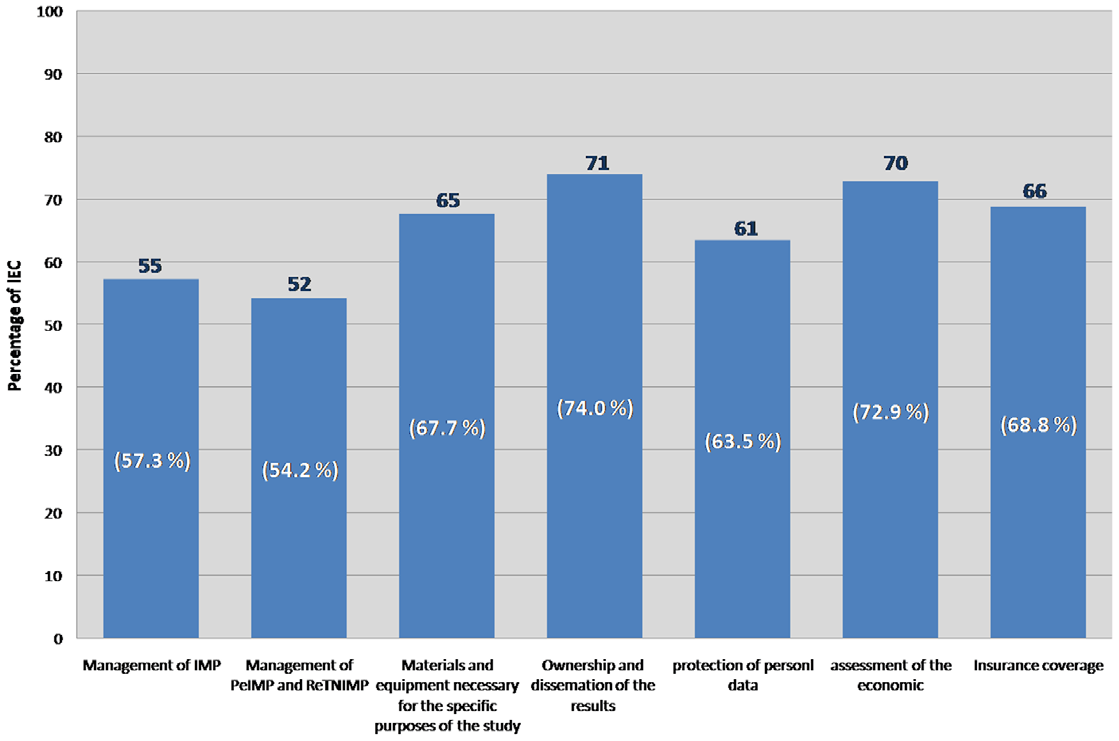
Documentation “centre-specific” from the Sponsor or CRO. IECs that consider the “centre-specific” documentation as necessary are 96 out of 131 that answered the survey.

The documentation considered as necessary for the evaluation by the IECs is mainly related to the management of Investigational Medicinal Product (IMP) and of Product Equivalent to IMP (PeIMP) and Regardless to Trial Non Investigational Medicinal Product (ReTNIMP), the materials and necessary equipment for the specific purposes of the study, the ownership of the results/publication and dissemination of results, the protection of personal data, and the assessment of the economic and insurance coverage (Figure 2).

### 3. “Centre-specific” Documentation Required of the Local Principal Investigator

One hundred and five (80.2%) IECs consider the ”centre-specific” documentation required from the PI as necessary. Among these, forty-three (41%) believe electronic submission of the ”centre- specific” documentation to be the most appropriate modality while fifty-six (53.4%) believe paper submission to be the most appropriate.

**Figure 3:**
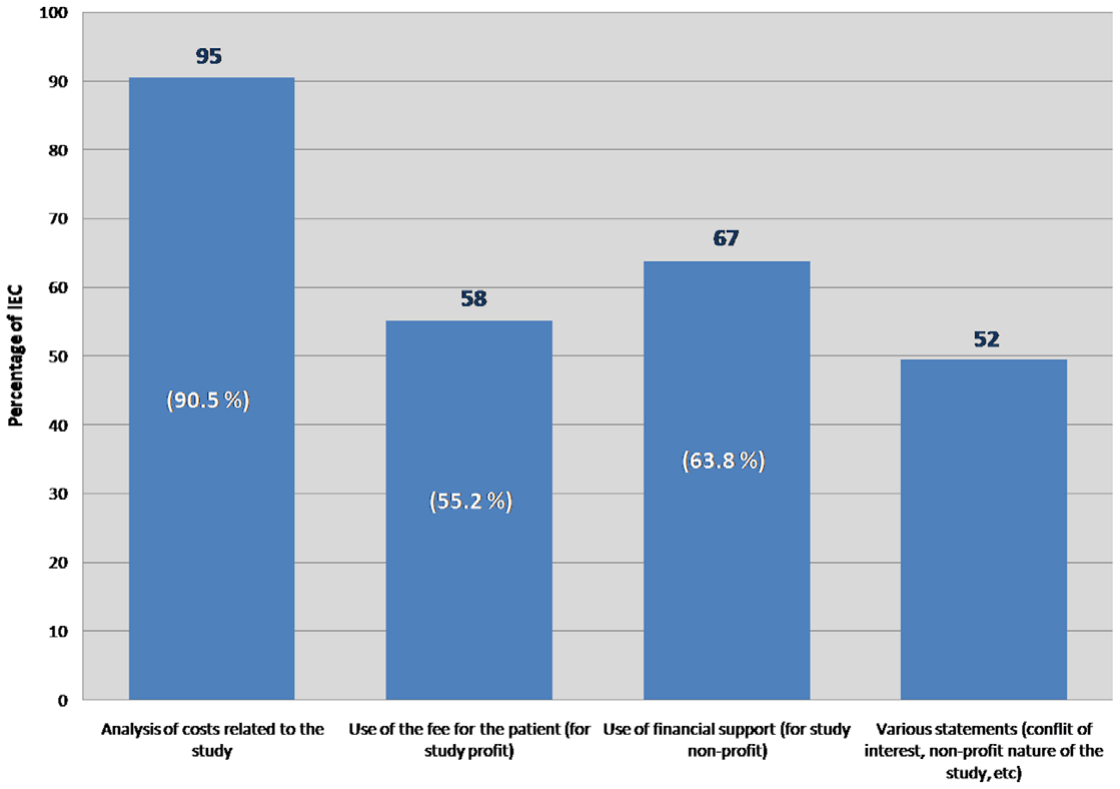
Documentation “centre-specific” from the local principal investigator. IECs that consider the “centre-specific” documentation as necessary are 105 out of 131 that answered the survey.

The documentation considered necessary for the evaluation by the IECs is mainly related to the analysis of costs of the study, the use of the fee per patient treated (for profit studies) and the use of financial support (for non-profit studies) and various statements (conflict of interest, non-profit nature of the study, etc.) (Figure 3).

### 4. “Single Document” Required in the Form of Statement from the PI/Sponsor by IECs

Based on the results of the first survey, a “single document” was prepared to establish, at the national level, the “minimum” elements required by IECs. The first survey showed that most of the information required by IECs was already included in the CTA form and supporting documents.

For this reason, two Forms were prepared to collect only information not already provided with the documentation required by MD-CTA.

In particular, Form 1 includes a section about the cost analysis of the study, a section about the use of funding prediction for the study, one on the involvement of nursing staff, one on the assumption of responsibility and on the conflict of interest statement by the PI.

Form 2 includes a section related to the provision of the IMP and its possible funding, and one regarding the involvement of the hospital pharmacy in preparing the IMP.

From April 19^th^, 2010 to May 14^th^, 2010, 125/131 IECs (95.4%) answered the second survey. Seventy-two (57.6%) consider Form 1 as an acceptable substitute to any other “centre-specific” documentation already in use, while thirty-three (26.4%) deemed some amendments necessary. In particular, difficulties emerged in the estimate of costs and the use of funds, and in the providing the extent of support staff to be employed in the study.

One hundred (80%) consider the proposed documentation on Form 2 as acceptable in substitution to any other “centre-specific” documentation already in use, while twelve (12%) deemed some amendments necessary.

The most critical one was linked to the provision of the IMP when used outside its marketing authorization in non-profit studies. While the Ministerial Decree 17/12/2004 [10] foresees that the “any additional expenditure, including the cost of the trial drug, required for the non-profit clinical trial, unless covered by ad hoc research funds, may be charged to the funds of health facilities”, not all have set up a *special* fund to cover the costs of the experimental drug.

## Discussion

Even though the MD-CTA entered into force on July 4^th^, 2008, most of the IECs in Italy still require “centre-specific” documentation, from both the Sponsor and the PI before issuing its opinion. For the Sponsor, such documentation is mainly related to the ownership, publication and dissemination of results, economic assessment, and insurance coverage. On the other hand, the additional documentation for the PI is mainly related to local feasibility assessment, the analysis study related costs and to use of the fee per patient treated (for profit studies), and to the use of financial support (for non-profit studies).

In addition, our survey reported that three of four and one of two IECs believe electronic submission to be the most appropriate way to send the “centre-specific” documentation, for the Sponsor and for the PI, respectively.

However, the electronic submission of “centre-specific” documentation will not streamline the process and reduce the time of activation of a clinical trial if the “centre-specific” documentation is not substantially reduced.

We firmly believe that a “single document”, in addition to reducing the variability of the “centre-specific” documentation, can reduce the activation time of the participating centres for multicentre clinical trials. It is noteworthy that more than 84% of IECs responding to the survey, consider the Forms to be an acceptable substitution to any other “centre-specific” documentation already in use.

The high variability of “centre-specific” documentation was largely due, to the high number of IECs. Despite the efforts to aggregate them, IECs remain too numerous, creating an unmanageable situation (especially concerning the acceptance/refusal of an opinion). In fact, compared with other European countries, Italy is an anomaly. There are more than 260 ethics committees in Italy, while in the United Kingdom they are 120, in Germany 50 and in France only 25 [7].

A large number of IECs certainly represents a form of democracy, but that does not automatically guarantee a better protection of the rights, safety and welfare of the subjects enrolled in the studies, and can actually lead to an immobilization of the system. It is reasonable to imagine that, within such a high number of IECs, many lack minimum structural requirements to work efficiently. In fact, our data shows that only 67% and 51% of IECs consider the structural requirements and the staff resources dedicated to the technical-scientific secretariat of the IECs as sufficient, respectively.

We believe that these committees play a role in the time required for activation of centres participating to multicentre clinical trials, and actually prevent the possibility of clinical investigators and their patients to participate in clinical trials, that in some cases might offer positive chances of treatment. In fact, the literature suggests that steps to develop and time to activate multicentre clinical trials are too extensive and highly variable, leading to an increase of the costs needed to open multicentre clinical trials [11], [12]. In particular, in many countries, critical delays are reported in obtaining regulatory approvals to initiate clinical trials: obtaining approval can take as long as 6 to 9 months [13], a time so lengthy it may be unethical [14].

According to the last Report of the National Monitoring Centre for Clinical Trials [15], in Italy, the average time for issuing the “single opinion” is decreasing nation-wide. An opposite trend has been experienced in the average time for issuing the acceptance/refusal of the single opinion, which was higher in 2010 than in previous years, and exceeding the foreseen time established by the Italian regulations. Routine or triggered GCP system inspections have been conducted by AIFA to fully understand the nature of IECs delays.

Moreover, a survey on the practice of ethics committees in 10 European countries, reported that in Italy harmonization and internal procedures guideline are lacking [16].

We believe that by establishing, at the national level, the documentation to be submitted to the IECs for the evaluation of a clinical trial and introducing the electronic submission as the only method of sending the documents, an important step could be made in order to “harmonize the practice of ethics committees” in Italy. We know that much still remains to be done.

In fact, one of the major problems encountered during the plenary meetings is how to send the Forms. While Form 2 can be sent in support of the documentation required by MD-CTA by *e-submission*, Form 1 cannot be sent in the same way as for structural problems OsSC (eg. exponential increase in the release of passwords access to the system for all the PI of the study; ease of error in the document relate to the study to be carried out, etc.).

Still, the forwarding in parallel of Form 1 would make difficult its tracking as well as any delay in submission of the same to technical-scientific secretariat could lead to a slowdown in the evaluation of the study by the local IEC, extending the time for issuing.

AIFA is in a key position facilitate clinical research by harmonizing the procedures concerning the approval of clinical trials in Italy, and reducing the time of activation of multicentre clinical trials. This survey is the first step in this direction.

Similar efforts could be undertaken to standardize the administrative procedures. Currently there are three different Competent Authorities according to the type of the study: AIFA for clinical trials with Advanced Therapy IMP; ISS for first-in-man use (phase I studies) and local Competent Authority for phase II, III, IV, and Bioequivalence/Bioavailability studies.

Another essential step is, therefore, aiming to have a single Competent Authority working together with the IEC in the coordinated assessment of the clinical trials to issue a Member State decision. AIFA is working towards this goal.
